# Insulin Pump in Difficult to Control Type 2 Diabetes: A Single Center, Five Years' Experience

**DOI:** 10.7759/cureus.3240

**Published:** 2018-08-31

**Authors:** Priyamvada Singh, Deepali Pandey, Nitin Trivedi

**Affiliations:** 1 Medicine, Ohio State University, Columbus, USA; 2 Internal Medicine, Saint Vincent Hospital, Worcester, USA; 3 Endocrinology, Saint Vincent Hospital, Worcester, USA

**Keywords:** type 2 diabetes mellitus, continuous subcutaneous insulin infusion, glycosylated hemoglobin, insulin pump, hba1c

## Abstract

Objective

Due to a progressive decline in beta-cell function, a considerable number of patients with type 2 diabetes mellitus (T2D) ultimately require multiple daily injections of large doses of insulin for glycemic control. Majority of studies have reported only short-term benefits of continuous subcutaneous insulin infusion (CSII) using an insulin pump in T2D. Our five-year follow-up data of CSII in T2D is one of the few studies showing persistent benefit in glucose control in this population.

Research design and methods

We did a chart review of patients treated with an insulin pump for five years. Inclusion criteria were: type 2 diabetes, 18–75 years of age, glycosylated hemoglobin (HbA1c) more than 6.5% (48 mmol/mol) on multiple doses of insulin (MDI > four injections per day) or more than 100 units of insulin/day, wide glycemic excursions, and intractable hypoglycemia. We identified a total of 13 patients. The primary endpoint was change in HbA1c from baseline to five years. We also reviewed the difference in weight, basal insulin requirements, hypoglycemia, and patient satisfaction questionnaire at one year. Exclusion criteria were: type 1 diabetes (T1D) and pregnancy.

Results

The HbA1c at five years was found to be 7.72% (61 mmol/mol) compared to a baseline of 8.89% (74 mmol/mol), p-value 0.0076. We did not find any increased risk of severe hypoglycemia, weight gain, and insulin requirement.

Conclusions

The beneficial effect of insulin pump persisted for five years of follow-up, suggesting it as a valuable treatment option for difficult to treat T2D.

## Introduction

Despite many anti-hyperglycemic agents, a considerable number of patients with type 2 diabetes mellitus (T2D), including those treated with basal-bolus insulin therapy, have sub-optimal glycemic control. Additionally, delivering large and multiple doses of insulin (MDI, more than four basal-bolus insulin injections per day) presents a therapeutic challenge. Continuous subcutaneous insulin infusion (CSII), on the other hand, is the continuous infusion of short-acting insulin via a needle or soft cannula under the skin using an electromechanical pump at a preselected basal rate with patients activated boosts with meals [1]. The advantage of CSII over MDI is that CSII mimics the baseline physiological insulin secretion of the body avoiding the wide glycemic excursions [2]. CSII is widely used in type 1 diabetes (T1D). However, its utility in T2D is not well established.

OpT2mise trial was a multi-centric, randomized controlled trial which compared insulin pump with MDI in 331 patients with poorly controlled T2D (baseline mean glycated hemoglobin (HbA1c) - 9% in both groups). HbA1c decreased by 1.1% versus 0.4% in the pump, and MDI groups respectively, p < 0·0001 [3]. Similar results were shown in other studies with a shorter duration of follow-up [4-9]. A recent study by Morera et al. showed a statistically significant decrease in HbA1c over nine years of follow-up with CSII when compared to MDI [9].

We present our real world five years’ experience of CSII in the difficult to control T2D population with a primary objective to assess the effect on HbA1c from baseline to five years, and secondary objectives of difference in weight, basal insulin requirements, hypoglycemia, and patient satisfaction questionnaire at one year.

## Materials and methods

We performed a retrospective analysis of patients treated with CSII at Saint Vincent Hospital, from the year 2005 to 2013. We found a total of 17 patients with the search term of “insulin pump, CSII, and type 2 diabetes”. Inclusion criteria for the study were: type 2 diabetes, 18–75 years of age, problems with glycemic control and/or insulin therapy (HbA1c more than 6.5% (48 mmol/mol), MDI > four injections per day, more than 100 units of insulin/day, wide glycemic excursions, and intractable hypoglycemia). Exclusion criteria were type 1 diabetes and pregnancy. Out of this, a total of 13 patients were selected for the final study based on the inclusion/exclusion criteria. All patients data were collected under protocol #1171, which was reviewed and approved by the Institutional Review Board at Saint Vincent Hospital.

The baseline characteristics are described in details below (Table 1).

**Table 1:** Baseline Characteristics Eleven subjects were on insulin aspart (100 units/ml), two on Humulin R U-500, two subjects were on metformin. OHA: Oral hypoglycemic agent. BMI: Body mass index (kg/m^2^).

Baseline characteristics	
Age (in years)	55 (43–71)
Sex	10 males, 3 females
Race	12 Caucasians, 1 African American
Insulin + OHA	2
Insulin only	11
BMI	34.5 (22–47)
Retinopathy	2
Nephropathy	2
Neuropathy	2

All patients were on MDI therapies with or without oral anti-diabetic medications before the initiation of CSII. The mean baseline HbA1c prior to CSII was 8.8923% (74 mmol/mol ± 1.15) (Range – 6.6% (49 mmol/mol) – 10.8% (95 mmol/mol)). One patient had HbA1c less than 7.0% (53 mmol/mol) and was switched to CSII because of recurrent nocturnal hypoglycemia. All patients used Medtronic Insulin pumps. Most of the subjects had some form of cardiovascular comorbidities (cerebrovascular accident, coronary artery disease, and peripheral vascular disease).

The primary endpoint was change in HbA1c from baseline to five years. In order to examine the short and long-term stabilization of HbA1c, we collected data at baseline, 3, 6, 9, 12 and 60 months. The frequency of measurement of the HbA1c level at the interval mentioned above was based on the clinical discretion of the endocrinologist. Secondary endpoints were changes in weight, basal insulin requirement, and severe hypoglycemia (glucose < 40 mg/dL or presence of neurologic dysfunction due to hypoglycemia requiring assistance) from baseline to one year. A ‘patient satisfaction questionnaire’ was analyzed at the end of one year and included ratings for physical health (concerning work or other regular activities), social activities, the flexibility of use, functions, and satisfaction with the insulin pump.

All patients received comprehensive education about insulin pump usage. Only two patients used continuous glucose monitoring. During the first eight weeks, the pump settings were adjusted every one to two weeks based on glucose readings on pump downloads. After that, the patients were encouraged to follow every two to three months. Only one endocrinologist performed the pump adjustments.

We calculated the mean of the available data and performed the statistical analyses with paired t-test using GraphPad InStat 3 software for the primary endpoint.

## Results

**Figure 1:**
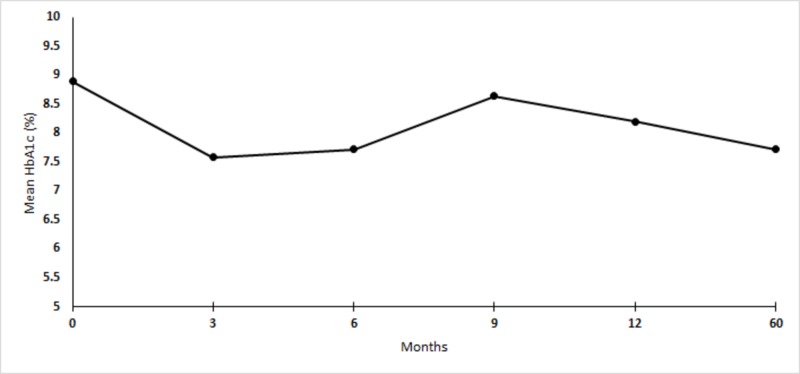
Mean HbA1c of 13 patients at baseline, 3, 6, 9, 12, 60 months.

**Figure 2:**
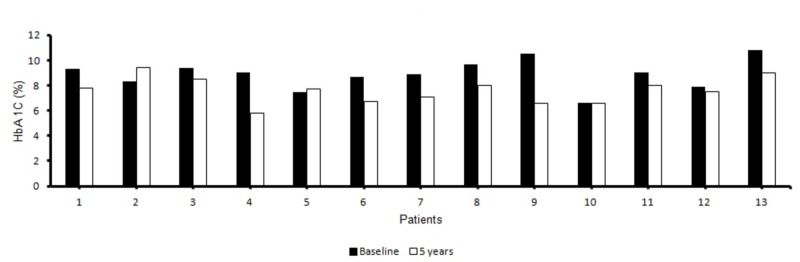
Mean HbA1c at the end of five years compared to baseline for all 13 patients.

Mean HbA1c at baseline, 3, 6, 9, 12, and 60 months were 8.89% (74 mmol/mol), 7.58% (59 mmol/mol), 7.72% (61 mmol/mol), 8.64% (71 mmol/mol), 8.20% (66 mmol/mol), and 7.7231% (61 mmol/mol), respectively (Figure 1). In our data, a sustained decrease in HbA1c was observed up to five years after initiation of CSII (Figure 2). Two-tailed p-value comparing baseline HbA1c, with five years was statistically significant (p = 0.0076). We did not find any episodes of severe hypoglycemia or hospitalization due to pump dysfunction during the five years of follow-up. One of the patients with HbA1C of 6.6 (49 mmol/mol) was started on CSII with continuous glucose monitoring for severe hypoglycemia. There were no episodes of severe hypoglycemia in him over five-year follow-up. However, he continued to have minor non-life threatening hypoglycemic episodes.

**Figure 3:**
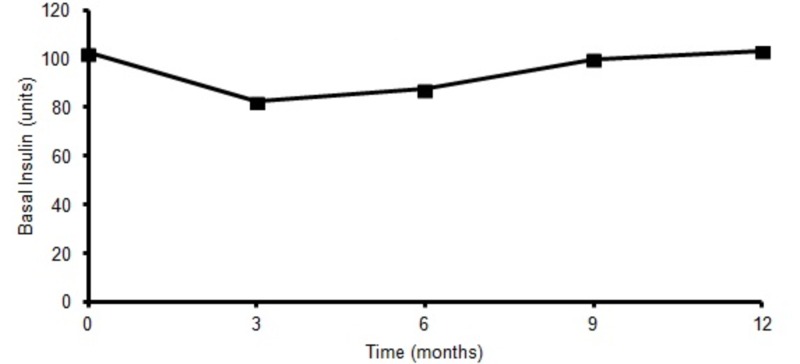
Basal insulin requirement at baseline, 3, 6, 9, 12 months.

**Figure 4:**
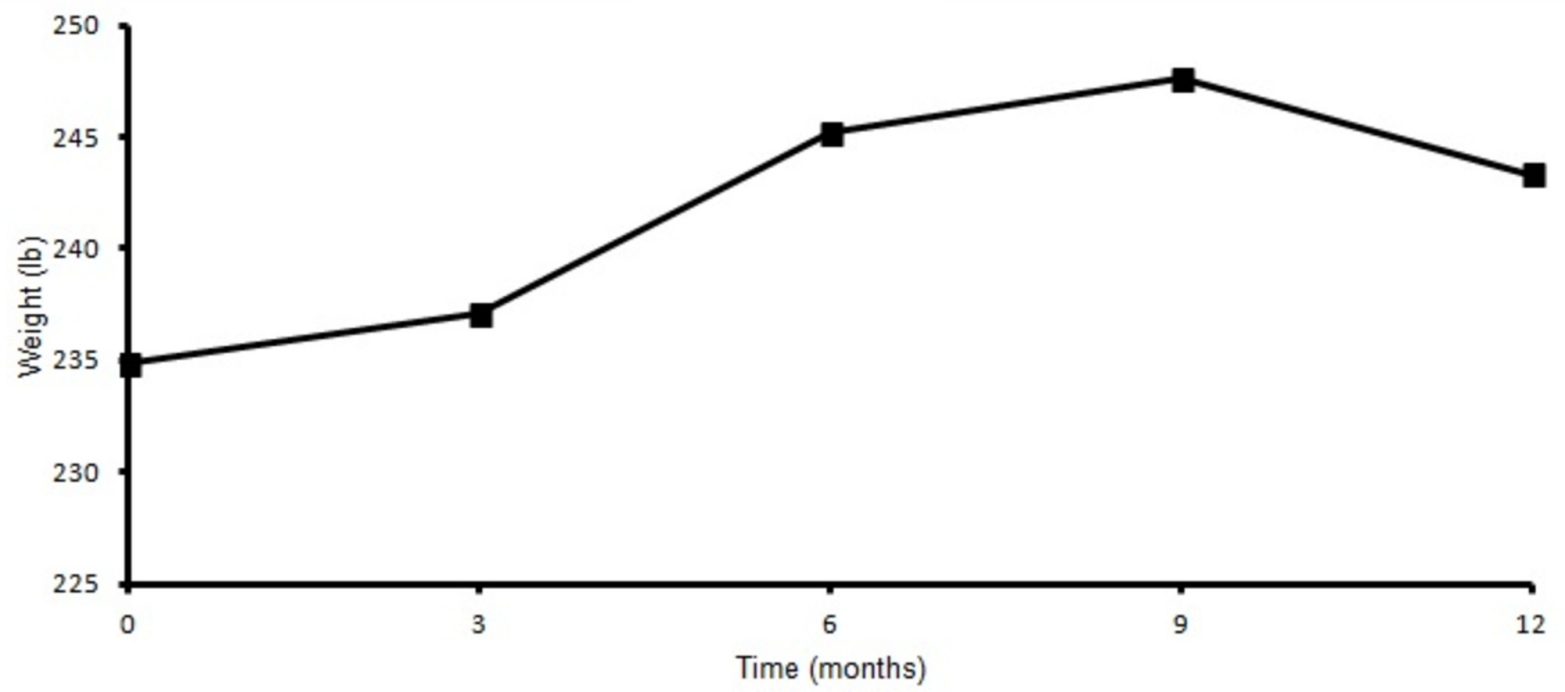
Weight at baseline, 3, 6, 9, 12 months.

Basal insulin requirement was 100 units at baseline and decreased to 80 units at three months but ultimately came to the baseline at the end of one year (Figure 3). Also, there was no significant weight gain at the end of one year (Figure 4). Nine out of 13 patients answered patient satisfaction questionnaire at the end of one year. On a scale of one to five, one being poor and five being excellent, the average score for CSII was four with regards to improvement in work, physical activity, social activity, and insulin pump performance compared to MDI. Interestingly, the oldest patient in our study was 71 years old with HbA1c of 9.3% at baseline (78 mmol/mol) which improved to 7.1% (54 mmol/mol) within one year. Encouragingly none of the patients switched to MDI from CSII.

## Discussion

In type 2 diabetes, there have been mixed results on CSII superiority over MDI with regards to glycemic control [3-9,10-13]. A few initial randomized controlled trials and meta-analyses did not show a statistically significant difference between CSII and MDI [10-13]. Whereas, the OpT2mise study demonstrated statistically significant HbA1c drop of 1.1% in the CSII group compared to 0.4% in MDI (p < 0.001) [3]. A more recently published study by Morera et al. showed a 1.3% decrease in HbA1c from baseline at the end of one year in a similar study population (p < 0.001) [9]. The HbA1c decline was maintained over nine years of follow-up (p-value < 0.05) [9]. Our data is reinforcing the results of these previous studies and demonstrated a sustained decrease of HbA1c over a five-year of follow-up study period.

Higher risk of severe hypoglycemia typically accompanies improved glycemic control. It was reassuring to note that the incidence of severe hypoglycemia did not increase at the end of one year of follow-up. Additionally, there were no reported hospitalizations for pump dysfunction or hyperglycemic emergencies in our research. These findings were similar to the previous studies [3-5,10-12].

Furthermore, in our study mean basal insulin requirement remained stable at the end of one year of CSII treatment (Figure 3). Interestingly, it did decrease initially at three and six months but was later stabilized to baseline requirement levels at the end of one year. Similar to our study, the OPT2mise trial showed a statistically significant decrease in insulin requirement at the end of six months [3]. On the contrary, studies with longer duration of follow-up did not show any statistical difference in mean insulin requirements [9]. This discrepancy in results between studies could be secondary to the fact that there is a progressive degradation of beta-cell in type 2 diabetes patients over a longer duration which is not captured during the initial follow-up phase [14].

Diabetes is a chronic progressive disease that significantly affects the quality of life of the patient. Multiple insulin injections are one of the main reasons for non-compliance in diabetes patients. Despite having a wide range of age variation, none of the patients discontinued CSII therapy in our study. The overall patient satisfaction score of 80% reflects acceptability, ease of administration, tolerability and improved lifestyle in this subset of the patient population. Our findings were similar to other studies which showed high satisfaction with CSII and low interference with daily activities [4,7,10,11].

Weight gain is a known complication of high insulin doses. In our study, the subjects had non-significant weight gain (Figure 4) at the end of one year period which is similar to previous literature [3,4,6-8,10-11].

The rate of type 2 diabetes increases with age. According to the ‘National Diabetes Statistics Report 2017’, the percentage of the population with type 2 diabetes is 25%, 17% and 4% in >65 years, 45–64 years, and 18–44 years, respectively [15]. We included a wide range of age group in our patient population to assess the efficacy, tolerability, and ease of administration of CSII in different age groups. Encouragingly, the oldest subject in our study was 71 years of age at the time of enrollment and was successfully able to understand and perform the operations of CSII system.

Despite published data on the safety and efficacy, CSII is not commonly used in T2D. In our clinical experience, CSII approval for T2D is met with relative resistance compared to T1D. This might be one of the reasons for non-familiarity of CSII with internists resulting in low utilization of CSII in T2D along with lack of proper insurance coverage, large out of pocket expense [16]. Our experience strengthens the concept that CSII is a safe, efficacious and durable option for T2D. Furthermore, CSII adjustments are much more straightforward in T2D compared with modifications in T1D.

Our study has a few limitations. One of the weaknesses is that the patient population primarily constituted of Caucasian males which decrease the generalizability of the study to other ethnicities. Other restrictions of the study are the small sample size and the retrospective design. One of the other limitations was that we created the patient satisfaction questionnaire based on our patient population, and it was not authenticated but has the advantage of the real world. Similar to other studies in this field we did not evaluate the effect on the cardiovascular outcomes, which is an area of future research.

## Conclusions

Despite being small-sized and retrospective, our study is a real-world experience of efficacy, safety and patient tolerability of CSII in difficult to control T2D patients and is thus noteworthy. The beneficial effect of CSII persisted for five years of follow-up, suggesting it as a valuable treatment option for this patient population. We hope these positive results will ease the insurance coverage of CSII for the T2D patient population too. Further studies would be needed to analyze the effect of the insulin pump on diabetes-related microvascular and cardiovascular complications.
